# Amyloid β-peptide oligomers, ryanodine receptor-mediated Ca^2+^ release, and Wnt-5a/Ca^2+^ signaling: opposing roles in neuronal mitochondrial dynamics?

**DOI:** 10.3389/fncel.2013.00120

**Published:** 2013-07-30

**Authors:** Andrea C. Paula-Lima, Cecilia Hidalgo

**Affiliations:** ^1^Faculty of Dentistry, Department of Basic and Communitarian Sciences, Universidad de ChileSantiago, Chile; ^2^Centro de Estudios Moleculares de la Célula, Universidad de ChileSantiago, Chile; ^3^Biomedical Neuroscience Institute, Universidad de ChileSantiago, Chile; ^4^Physiology and Biophysics Program, Faculty of Medicine, Institute of Biomedical Sciences, Universidad de ChileSantiago, Chile

Alzheimer's disease (AD) is the most common form of dementia in the elderly (Querfurth and Laferla, 2010). Recent evidence indicates that soluble neurotoxic Aβ oligomers (AβOs) play a causative role in AD pathogenesis, since they accumulate in the brain of affected individuals and bind specifically to excitatory synapses, prompting changes in their composition, shape, and density (Paula-Lima et al., 2013). These toxic effects presumably underlie the loss of neuronal connectivity characteristic of AD (Ferreira and Klein, 2011). In primary hippocampal neurons, AβOs induce Ca^2+^ entry through N-Methyl-D-aspartate (NMDA) receptors and promote reactive oxygen species (ROS) generation (De Felice et al., 2007). The ensuing increase in postsynaptic Ca^2+^ and ROS levels promotes Ca^2+^ release from the endoplasmic reticulum (ER) via joint stimulation of the ER redox-sensitive ryanodine receptor (RyR) channels (Paula-Lima et al., 2011). The resulting unusually long-lasting Ca^2+^ signals prevent the dendritic spine remodeling induced by brain-derived neurotrophic factor, among other effects (Paula-Lima et al., 2011). Additionally, a previous report indicated that AβOs-induced Ca^2+^ release causes ER stress, oxidative damage, and cell death (Resende et al., 2008).

Abnormal mitochondrial function likely plays an important role in AD (Lin and Beal, 2006; Cho et al., 2010; Manji et al., 2012; Itoh et al., 2013). Current studies have demonstrated the existence of mitochondrial-ER contact sites, originating microdomains of localized Ca^2+^signal generation (Csordas et al., 2010). Mitochondria operate either as a barrier Ca^2+^ buffer or as facilitating factors in the spreading of Ca^2+^ signals to the nucleus (Alonso et al., 2006). Mitochondria are highly dynamic structures, which in live neurons divide, fuse, and move within axons and dendrites (Cheng et al., 2010). We have reported that the long-lasting Ca^2+^ signals generated by AβOs in primary hippocampal neurons disrupt mitochondrial network structure; suppressing RyR activity by pre-incubation with inhibitory ryanodine prevents AβOs-induced mitochondrial fission, indicating that this process requires the RyR-mediated Ca^2+^ signals generated by AβOs (Paula-Lima et al., 2011). Direct activation of RyR-mediated Ca^2+^ release by the RyR agonist 4-chloro methyl cresol promotes mitochondrial network fragmentation in primary hippocampal neurons, further indicating that RyR activation promotes mitochondrial fission (Sanmartin et al., 2012). Of particular relevance in this regard are recent reports showing that increased mitochondrial network fission occurs in neurodegenerative diseases and diabetes (Yoon et al., 2011; Itoh et al., 2013).

The work by Silva-Alvarez and colleagues confirms that RyR inhibition with ryanodine prevents the alterations in mitochondrial morphology induced by AβOs. They also show that pre-incubation of primary hippocampal neurons with AβOs plus thapsigargin, an irreversible inhibitor of the sarco/endoplasmic reticulum Ca^2+^-ATPAse (SERCA), causes irreversible mitochondrial fragmentation, suggesting that both RyR and SERCA contribute to the loss of Ca^2+^ homeostasis induced by AβOs (Silva-Alvarez et al., 2013). An alternative explanation would be, however, that the permanent ER depletion produced by SERCA inhibition with thapsigargin contributes to the irreversible mitochondrial fragmentation produced by AβOs. According to Silva-Alvarez et al. (2013), the mitochondrial fragmentation promoted by AβOs may involve Ca^2+^-dependent activation of signaling pathways that promote mitochondrial fission, as detailed below.

An important new finding presented by Silva-Alvarez et al. (2013) is that activation of Wnt signaling by the non-canonical Wnt-5a ligand prevents the RyR-mediated mitochondrial fragmentation induced by AβOs. Previous work from these and other authors have implicated Wnt signaling in synaptic plasticity, in modulation of long-term potentiation (LTP) (Cheng et al., 2010; Cerpa et al., 2011) and in neuroprotection (Toledo et al., 2008). In their current report, Silva-Alvarez and colleagues show that activation of Wnt-5a-mediated signaling protects neurons from AβOs toxicity, preventing the increased mitochondrial fission and the Bcl-2 exposure to the mitochondrial outer membrane caused by AβOs (Silva-Alvarez et al., 2013). Based on their unpublished results, these authors further propose that non-canonical Wnt signaling induced by Wnt-5a inhibits mitochondrial fission via a mechanism that involves Ca^2+^ release from the ER. To explain their results, Silva-Alvarez et al. (2013) propose that Wnt-5a binding to its Frizzled receptor activates Dishevelled, which in turn would activate a signaling cascade involving a trimeric G protein, phospholipase C (PLC), and generation of inositol 1,4,5-trisphosphate (IP_3_), which increases intracellular Ca^2+^ by promoting IP_3_-receptor mediated Ca^2+^ release from the ER. Through Ca^2+^-induced Ca^2+^ release, the ensuing Ca^2+^ increase would promote RyR-mediated Ca^2+^ release, generating Ca^2+^ signals that activate Ca^2+^-dependent kinases such as PKC and CaMKIα, or the phosphatase calcineurin, which would affect mitochondrial dynamics via activation of Dynamin-related protein (Drp1), a protein critically involved in mitochondrial fission (Smirnova et al., 2001; Qi et al., 2011). In fact, some evidence implicates activation of these enzymes by AβOs-generated Ca^2+^ signals. Thus, inhibition of the PKC pathway reduces the cell death induced by AβOs (Kriem et al., 2005) while inhibition of a pathway engaging CAMKK2-AMPK-Tau prevents the synaptotoxic effects of AβOs (Mairet-Coello et al., 2013); additionally, calcineurin activation mediates the synaptic defects and memory disruption induced by AβOs (Reese and Taglialatela, 2011). Moreover, we reported that AβOs promote Drp-1 translocation to the mitochondria in primary hippocampal neurons; this translocation does not occur following inhibition of RyR-mediated Ca^2+^ release (Paula-Lima et al., 2011). Therefore, Ca^2+^ signaling, and in particular RyR-mediated Ca^2+^-release, plays a critical role in the fragmentation of mitochondrial network induced by AβOs. Overall, the work of Silva-Alvarez et al. (2013) indicates that Ca^2+^ release from the ER lies downstream of the non-canonical Wnt-5a ligand binding to its receptor.

Although AD pathogenesis has been extensively studied over the last 100 years, no curative or preventive treatments are available at present for effective patient treatment. Many efforts have been made to establish new targets to counteract the deleterious effects of AβOs on neuronal function. The beneficial effects of Wnt-5a signaling against the mitochondrial network damage induced by AβOs reported by Silva-Alvarez et al. (2013) raises a new approach to counteract the aberrant Ca^2+^signals induced by AβOs. Thus, the Wnt-5a signaling pathway might constitute a possible target for the development of new therapeutic treatments for AD.

## Abbreviations

AD: Alzheimer's disease
AMPK: AMP-activated protein kinase
Aβ: amyloid-β peptide
AβOs: difusible Aβ oligomers
CaMKIα: calcium/calmodulin-dependent protein kinase Iα
CAMKK2: calcium/calmodulin-dependent protein kinase 2
Drp1: Dynamin-related protein
ER: endoplasmic reticulum
IP3: inositol 1,4,5-trisphosphate
LTP: long-term potentiation
NMDA: N-methyl-D-aspartate glutamate
PKC: protein kinase C
PLC: Phospholipase C
ROS: reactive oxygen species
RyR: Ryanodine Receptor
SERCA: sarco/endoplasmic reticulum Ca^2+^-ATPase.
